# Uncoupling of Microvascular Blood Flow and Capillary Density in Vascular Cognitive Impairment

**DOI:** 10.3389/fneur.2019.01268

**Published:** 2019-12-03

**Authors:** Chenxing Eleana Zhang, Julie Staals, Robert Jan van Oostenbrugge, Hans Vink

**Affiliations:** ^1^Department of Neurology, Maastricht University Medical Center, Maastricht, Netherlands; ^2^CARIM and MHeNs, Maastricht University Medical Center, Maastricht, Netherlands; ^3^Department of Physiology, Maastricht University Medical Center, Maastricht, Netherlands

**Keywords:** cerebral small vessel disease, vascular cognitive impairment, capillary dysfunction, capillary density, sublingual intravital microscopy

## Abstract

Cerebral small vessel disease (cSVD) plays an important role in dementia and is a major cause for vascular cognitive impairment (VCI). Recent studies hypothesized that capillary dysfunction including reduction of capillary patency, rather than a flow-limiting pathology is crucial in cSVD. As cSVD is considered a systemic microvascular disease, we examined sublingual microvascular blood flow and capillary density in patients with VCI and controls. Fifteen patients with VCI due to cSVD and 15 controls underwent intravital microscopy of the sublingual microvessels. Microvascular blood flow and capillary density in high and low flow areas were determined for each participant. Flow-density coupling was examined by determining the ratio of density changes to flow changes, and the ratio of feed vessel red blood cell (RBC) velocity to capillary RBC velocity. These were compared between VCI and controls. In healthy controls, capillary density increased proportionally with feed vessel blood flow increase. In patients with VCI, no increase of capillary density was observed. Moreover, increase of feed vessel RBC velocity led to significant increase of capillary RBC velocity in VCI, whereas in controls, the capillary RBC increased only slightly. Flow-density coupling differed significantly between VCI and controls, also after correcting for age and hypertension. Our findings suggest uncoupling of microvascular blood flow and capillary density in patients with VCI. This uncoupling may impair oxygen and nutrients exchange when blood flow increases in response to increased metabolic demand, ultimately leading to tissue damage.

## Introduction

Cerebral small vessel disease (cSVD) is a disorder of the small arteries and veins in the brain and is considered the most important cause of vascular cognitive impairment (VCI) (1, 2). The exact pathophysiological mechanisms of cSVD and VCI are still to be elucidated.

It has been hypothesized that changes in cerebral perfusion play a role in cSVD and development of VCI (2, 3). However, findings are inconsistent: some studies reported lower cerebral perfusion in patients with cSVD and VCI compared with controls, while others found similar or higher perfusion values (4, 5). These inconclusive findings on the role of cerebral perfusion may be due to technical limitations in detecting subtle changes in microvascular blood flow as most techniques measure large vessel perfusion or are contaminated by large vessel influences, and furthermore, microvascular flow changes are probably more complex and dynamic.

Recently, it has been suggested that capillary dysfunction and disrupted capillary patency rather than flow reduction are essential mechanisms in cSVD (6). Brain tissue demand for oxygen and glucose is high and changes dynamically with local increases in metabolic activity. To match nutrient delivery to temporal changes in local metabolic need, it is essential that increases in microvascular blood flow are coupled to increase in the number of capillary blood vessels that are perfused with blood (from now on referred to as perfused capillary density). Changes in perfused capillary density are essential to adjust microvascular exchange surface area to local metabolic need (7). Uncoupling of perfused capillary density from microvascular flow may contribute to inadequate oxygen and nutrients delivery, despite an intact flow, and may eventually cause brain parenchymal damage as is seen in cSVD.

In the current study, we aim to determine coupling of microvascular blood flow and perfused capillary density in patients with VCI due to cSVD and in healthy controls. This was addressed in the sublingual vasculature which can easily be accessed and imaged, and has been shown to be involved in cSVD (8, 9).

## Materials and Methods

### Study Population

We included male patients with mild VCI due to cSVD—hence referred to as VCI—and healthy controls from the Memory Clinic and outpatient clinic of the Department of Neurology, of Maastricht University Medical Center and Zuyderland Hospital, The Netherlands, between April 2013 and December 2014 (10). Criteria of mild VCI were met when patients had (1) subjective complaints of cognitive functioning, and (2) objective cognitive impairment in at least one cognitive domain at neuropsychological testing, and (3) a Clinical Dementia Rating of ≤ 1 and a Mini Mental State Examination score of ≥20, and (4) vascular lesions on brain MRI that suggest a link between the cognitive deficit and cSVD (11): moderate to severe white matter hyperintensities (WHM; Fazekas score deep > 1 and/or periventricular > 2), or mild WMH (Fazekas score deep = 1 and/or periventricular = 2) combined with lacune(s) and/or microbleeds (12).

Healthy controls were defined as participants with no overt cerebrovascular diseases (TIA, stroke) and no cognitive impairment. Most of them had lumbar radicular syndrome or mononeuropathies. Additional exclusion criteria for all participants included neurodegenerative diseases, multiple sclerosis, epilepsy, systemic inflammatory diseases, alcohol abuse, and psychiatric disorders (10).

Characteristics of all participants were recorded including age, sex, and the presence of cardiovascular risk factors including hypertension (history of hypertension and/or use of blood pressure lowering drugs), hypercholesterolemia (history of hypercholesterolemia and/or use of statin), diabetes mellitus (history of diabetes mellitus or use of blood sugar lowering drugs), smoking (current smoking), and Body Mass Index (BMI: current weight of the subject by the square of the current length).

### Standard Protocol Approvals, Registrations, and Patient Consents

The Medical Ethical Committee of the Maastricht University Medical Center approved the study. All participants were included after written informed consent. The study is registered at www.trialregister.nl (NTR number NTR3786).

### Structural Magnetic Resonance Imaging

All participants underwent structural brain MR imaging (3.0 Tesla). T2-weighted FLAIR sequence was used for detection of WMH. Subsequently, a semi-automated segmentation tool was used to determine the WMH volume (13).

### Imaging of Sublingual Microvessels

Intravital microscopic recordings of sublingual microvessels were collected with a digital clinical video camera (KK Technology Ltd.) connected to a laptop based automated video acquisition and image analysis system (GlycoCheck BV) (14). A series of 10–20 short video recordings (<2 s long) were collected for each individual in order to obtain at least 3,000 vascular segments ranging from 5 to 25 μm in diameter. Vessels were automatically grouped in diameter classes and vessels with diameters between 4.5 and 5.5 μm were defined as capillaries, while vessels with diameters 8.5–9.5 μm were defined as feed vessels.

### Blood Flow Measurements

Feed vessel blood flow was measured in each video recording by adding the flows of all the individual feed vessels of that video recording, then divided by video surface area to normalize blood flow to tissue surface area. Blood flow of a given feed vessel was calculated by multiplying feed vessel red blood cell (RBC) velocity times feed vessel red cell content times feed vessel cross-sectional area.

For analysis of the effect of microvascular blood flow on capillary density, spontaneous variability of flow during the data recording allowed for selection of 2 videos per individual with low and high blood flow, respectively. Selection of the high and low flow states were based on visual inspection of the blood flow levels in the feeding blood vessels. From the list of available videos, the first available high flow video and the first available low flow video were selected and subsequent measurement of RBC velocities and flow levels in the feed vessels confirmed that the chosen videos represented similar levels of high flow and low flow in VCI patients and controls.

### Capillary Density

Capillary density is defined as the number of capillaries containing red cells per video, normalized to tissue surface area. All automatically detected red cell containing capillaries were counted in the low- and high flow video recording of each individual in order to test for the effect of changes in microvascular blood flow on capillary density.

### Coupling of Microvascular Flow and Capillary Density

Perfused capillary density should increase when microvascular blood flow increases to secure adequate metabolic exchange. To examine this coupling mechanism, we determined in each individual:

- Capillary density in the low and high flow videos.
- Ratio of change in density (i.e., the density in the high flow state minus the density in the low flow state) to change in flow (i.e., high flow minus low flow).
- Ratio of feed vessel RBC velocity to capillary RBC velocity. If the capillary density increases when microvascular blood flow increases, the relative increase in capillary RBC velocity will be smaller than the relative increase in feed vessel red cell velocity. This secures adequate oxygen and nutrients exchange. A larger ratio at a higher level of microvascular blood flow indicates a better coupling. An advantage of this way of analyzing capillary velocity instead of density is that capillaries containing stagnant RBCs are not taken into account, as these capillaries do not contribute to nutrient exchange.

### Statistical Analysis

Independent student *t*-test and chi-square test were used for comparing characteristics between patients with VCI and controls. The sublingual microvascular blood flow and capillary density were examined in high and low flow videos, and compared between patients with VCI and controls using independent and paired student *t*-test. The ratio of density to flow change and the ratio of feed vessel to capillary RBC velocity were calculated, and compared between patients with VCI and controls. In multivariable analyses, we used the ratio as dependent variable, and group, age, and hypertension as independent variables. All statistical analyses were performed using commercial software (SPSS 22). Statistical significance was inferred at *p* < 0.05.

## Results

We included 15 male patients with VCI and 15 male controls. Characteristics of these participants are presented in Table 1. Patients with VCI were significantly older and more of them had hypertension compared to healthy controls.

**Table 1:** Characteristics of patients with VCI and healthy controls.

	**VCI *N* = 15**	**Controls *N* = 15**
Age, years (SD)^*^	76 (7)	66 (12)
Hypertension (%)^*^	12 (80)	4 (27)
Hypercholesterolemia (%)	9 (60)	6 (40)
Diabetes Mellitus (%)	5 (33)	3 (20)
Current smoking (%)	3 (20)	1 (7)
BMI kg/m^2^ (SD)	26 (5)	26 (3)
WMH volume (SE)^*^	0.017 (0.003)	0.007 (0.004)

*VCI, Vascular Cognitive Impairment; BMI, Body Mass Index; WMH volume, white matter hyperintensities volume normalized to intracranial volume*.

* *p < 0.05 comparing VCI patients with controls*.

### Microvascular Blood Flow

**Figure 1:**
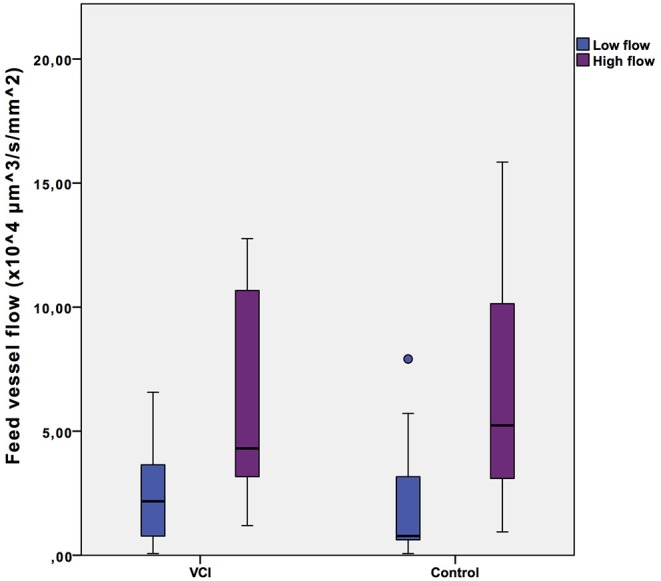
Intra-individual feed vessel flow range. Box plots with median, interquartile range (filled rectangles), and full range. VCI, patients with vascular cognitive impairment.

Microvascular blood flow as measured in a selected low flow and a high flow video of each individual in the group of healthy controls and in the group of patients with VCI is shown in Figure 1. The intra-individual differences between low flow and high flow were similar for healthy controls (2.1^*^10^4^ vs. 6.7^*^10^4^ μm^3^s^−1^mm^−2^, *p* = 0.001) and patients with VCI (2.5^*^10^4^ vs. 6.5^*^10^4^ μm^3^s^−1^mm^−2^, *p* = 0.003).

### Capillary Density

**Figure 2:**
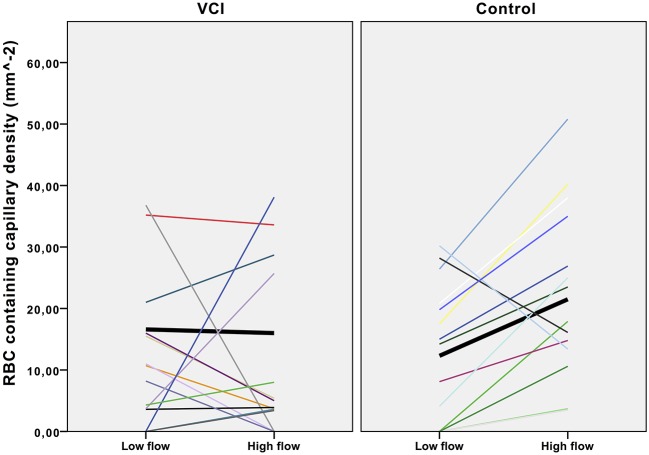
Effect of flow increase on intra-individual capillary density. Scatterplot with trend lines. VCI, vascular cognitive impairment. Each line represents the density change of one participant. Black lines in bold represent the mean density change.

Density of capillaries containing red cell was determined in a low flow video and a high flow video for each individual (Figure 2). As Figure 2 shows, capillary density increased in the high flow video compared with the low flow video in almost all healthy controls, whilst this increase was absent in patients with VCI. In healthy controls, capillary density was significantly lower in the low flow than in the high flow videos (mean 12.3 mm^−2^ vs. 21.5 mm^−2^ respectively, *p* = 0.009). Capillary density was identical in the low flow and high flow video of patients with VCI (mean 16.6 vs. 16.0 respectively, *p* = 0.89).

### Coupling of Microvascular Blood Flow and Capillary Density

The average ratio of capillary density change to microvascular flow change differed significantly between patients with VCI and healthy controls, with a negative ratio for patients with VCI and a positive ratio for healthy controls (mean −0.05 vs. 0.04, respectively; *p* = 0.02). In a multivariable analysis with correcting for age and hypertension, the association between ratio and group remained significant (β = 0.59, *p* = 0.009).

**Figure 3:**
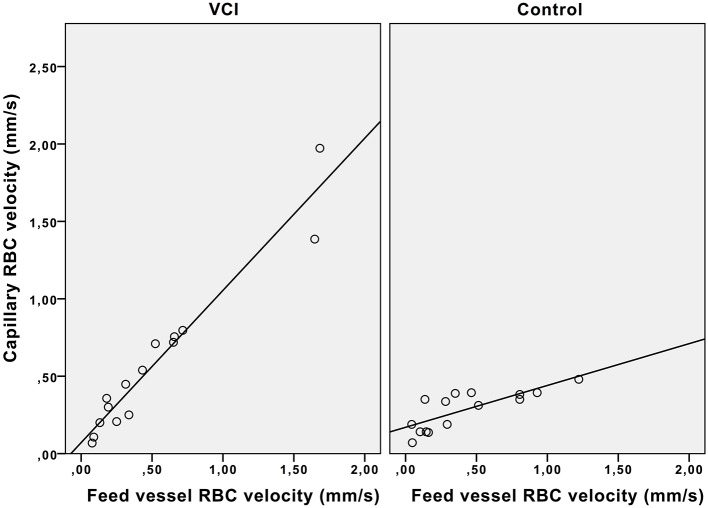
Effect of feed vessel RBC velocity increase on capillary RBC velocity. Scatterplot with trend lines. VCI, vascular cognitive impairment; RBC, red blood cell.

Figure 3 shows that in controls, the capillary RBC velocity increases only mildly with increase of feed vessel RBC velocity, whilst in patients with VCI this increase is much larger. The average ratio of feed vessel RBC velocity to capillary RBC velocity was also significantly different between patients with VCI and healthy controls (0.89 vs. 1.31, respectively, *p* = 0.04). In multivariable analysis with correction for age and hypertension, the association between ratio and group remained significant (β = 0.54, *p* = 0.01).

## Discussion

In this study, we aimed to examine the coupling of spontaneous variability in microvascular blood flow and capillary density in sublingual microvessels in patients with VCI. Many studies have demonstrated that microvascular blood flow in healthy individuals show spontaneous, temporal variability, related to the frequency of breathing, heart rate and variability of endothelial release of nitric oxide and other vasoactive substances (15–17). In this study, 10–20 videos of sublingual microvascular blood flow were collected within a 2–5 min time window and the 2-s duration of the individual videos is of appropriate length to capture different flow states within the 2–5 measurement duration.

Whereas, an earlier study already showed that the perfused sublingual capillary density was significantly lower in patients with cSVD than in healthy controls (9), we went a step further and examined the coupling between blood flow and functional capillary density. Our results underline that microvascular dysfunction in cSVD patients should be regarded as a reduction of capillary adaptation capacity: in patients with VCI, the capillary density does not increase significantly with increase of microvascular blood flow and capillary RBC velocity increases significantly when feed vessel RBC velocity increases. In contrast, healthy controls show an increase of capillary density with increase of microvascular blood flow, and a relatively constant capillary RBC velocity with increase of feed vessel RBC velocity.

Our data demonstrate uncoupling of capillary density and microvascular blood flow in individuals with VCI, suggesting a failure in optimizing exchange conditions during increased microvascular blood flow. The observed absence of capillary density increase when blood flow increases in patients with VCI will result in a reduced exchange capacity. This phenomenon observed at the level of the sublingual microvessels may also occur in the brain. Earlier sublingual microvascular measurements have shown significant differences between patients with cSVD, especially those with extensive WMH, and controls (8).

Our finding is in line with a recent review that proposed an important role of capillary dysfunction, in the pathophysiology of cSVD. (6) Although different mechanisms have been proposed on the regulation of the number of blood-perfused capillaries during changes in microvascular blood flow, the exact mechanisms have not yet been unraveled (18). Yet, the endothelium is likely to play a crucial role: disruption of endothelial cells signaling pathways has been shown to lead to significant disruptions of vascular control of the capillaries, resulting in impairment of exchange capacity (19). Animal models suggest that pericytes dysfunction contributes to this capillary shunting (20). Pericytes are glia cells that surround the capillary wall. Their contractile ability initiates subtle dilation or constriction of the capillaries and plays a pivotal role in regulating local flow on the capillary level.

Strength of our study is that we examined the blood flow of micro vessels with an established method in strictly phenotyped patient group. Studies on microvessels will provide more insights in the pathophysiology of cSVD compared to most perfusion studies that looked at, or had input from larger vessel blood flow.

This study should be considered a small proof of concept study and has limitations. A limitation is that the sublingual circulation does not necessarily reflect brain microvascular function. However, this current study does reveal a difference at the level of capillary blood flow control in patients with VCI compared with controls. This is a finding consistent with many other studies demonstrating that sublingual assessment of microvascular function allows identification of systemic microvascular vulnerability in many different patient groups including patients with diabetes, kidney disease, heart disease, stroke, and systemic inflammatory challenges like sepsis (8, 21–24). Additional studies need to test whether the observed sublingual uncoupling of microvascular blood flow and capillary density extends to the brain, but for now, our findings are in line with the concept that cSVD is a systemic endotheliopathy (25, 26). Another limitation is that our study was performed only in male participants. Non-published observations of sublingual microvascular measurements have shown strongly variable values in women compared to men. More studies are needed on reproducibility of sublingual intravital microscopy as earlier studies have shown different results (27–29). Furthermore, we need studies with lager sample sizes to confirm our results.

In conclusion, despite the small sample size in this proof of concept study, we have demonstrated uncoupling of sublingual microvascular blood flow and capillary density in patients with VCI. Additional studies are needed to confirm our findings and to determine whether the extent of this capillary dysfunction is also related to the severity of cSVD, such as the radiological extent of white matter hyperintensities and cognitive function. This may bring us to a better understanding of the pathophysiology of cSVD and its clinical consequences.

## Data Availability Statement

The datasets generated for this study are available on request to the corresponding author.

## Ethics Statement

This study was carried out in accordance with the recommendations of the Medical Ethical Committee Maastricht University Medical Center with written informed consent from all subjects. All subjects gave written informed consent in accordance with the Declaration of Helsinki. The protocol was approved by the Medical Ethical Committee Maastricht University Medical Center.

## Author Contributions

CZ: study concept and design, acquisition of data, analysis and interpretation of data, and writing manuscript. JS and RO: study concept and design, interpretation of data, and critical revision of manuscript. HV: study concept and design, analysis and interpretation of data, study supervision, and critical revision of manuscript.

### Conflict of Interest

HV is CSO of GlycoCheck BV. The remaining authors declare that the research was conducted in the absence of any commercial or financial relationships that could be construed as a potential conflict of interest.
